# Sociodemographic and Clinical Determinants of Fatigue in Multiple Sclerosis

**DOI:** 10.3390/life13112132

**Published:** 2023-10-29

**Authors:** Smaranda Maier, Zoltán Bajkó, Ruxandra Roșescu, Laura Bărcuțean, Emanuela Sărmășan, Septimiu Voidăzan, Rodica Bălașa

**Affiliations:** 1 Ist Neurology Clinic, Emergency Clinical County Hospital Targu Mures, 540136 Targu Mures, Romania; smaranda.maier@umfst.ro (S.M.); laura.barcutean@umfst.ro (L.B.); sarmasan.emanuela@gmail.com (E.S.); rodica.balasa@umfst.ro (R.B.); 2 Department of Neurology, ‘George Emil Palade’ University of Medicine, Pharmacy, Science, and Technology of Targu Mures, 540136 Targu Mures, Romania; 3 Department of Pediatric Neurology, Clinical Emergency Hospital for Children, 400394 Cluj-Napoca, Romania; ruxandra.rosescu@gmail.com; 4 Department of Epidemiology, ‘George Emil Palade’ University of Medicine, Pharmacy, Science, and Technology of Targu Mures, 540136 Targu Mures, Romania; septimiu.voidazan@umfst.ro

**Keywords:** multiple sclerosis, fatigue, Modified Fatigue Impact Scale, Beck Depression Inventory-II

## Abstract

Fatigue is the most common and disabling symptom in patients with multiple sclerosis (PwMS), representing one of the main determinants of reduced quality of life among PwMS due to its interference with social activities and work capacity. This study aimed to identify the sociodemographic determinants of fatigue in a cohort of 150 PwMS and 100 healthy controls (HCs). Fatigue was assessed using one of the most suitable and appropriate tools for measuring the degree of fatigue: the Modified Fatigue Impact Scale (MFIS). By comparing the median scores for the MFIS, we observed that the PwMS group had significantly higher MFIS scores than the HCs (*p* = 0.0001). In PwMS, MFIS scores correlated positively with age, total number of relapses, total disease duration, disability status, and Beck Depression Inventory-II score and negatively with cognitive performance. Patients with relapsing-remitting MS had significantly lower fatigue levels than those with secondary progressive MS (*p* = 0.0010). Fatigue levels were significantly lower among male than female PwMS (*p* = 0.0120). Other determinant factors of fatigue in our study proved to be the marital and occupational status, as well as the presence of children, but in a linear multivariate regressions analysis with MFIS score as the dependent variable, the fatigue levels were influenced only by sex, occupational status, marital status, children status, and BDI-II test results. Considering the significant impact of fatigue on the quality of life of PwMS, clinicians must diagnose fatigue as early as possible, identify its modifiable determinants, and manage it effectively to increase their quality of life.

## 1. Introduction

Multiple sclerosis (MS) is a chronic inflammatory, demyelinating, and neurodegenerative disease of the central nervous system (CNS). It is the most common cause of non-traumatic disability in young adults, affecting > 2.5 million worldwide [1,2,3].

MS is a highly heterogeneous disease, encompassing variations in pathophysiological processes, immunological changes, responses to treatment, and clinical manifestations. Several phenotypes of its clinical evolution have been described. In approximately 85% of cases, MS begins with episodes characterized by new neurological signs and symptoms affecting the CNS or the worsening of pre-existing symptoms. These episodes typically exhibit complete recovery under corticosteroid treatment followed by periods of remission, a condition known as relapsing-remitting MS (RRMS). After approximately 20 years of disease progression, approximately 60–70% of patients with an initial relapsing MS course will convert to secondary progressive MS (SPMS), defined retrospectively by gradual worsening disability with or without acute clinical exacerbations. Fewer patients will undergo a progressive evolution of neurological deficits from MS onset, known as primary-progressive MS (PPMS). PPMS and SPMS are part of the progressive MS phenotypic spectrum [4,5].

While the severity of MS was initially evaluated solely based on the degree of physical disability (motor deficit, presence of cerebellar signs, signs of brain stem damage, sphincter dysfunction, decreased visual acuity, and sensitivity disorders), in the last two decades, physicians have begun paying closer attention to “silent” non-motor manifestations of MS, such as fatigue, mood disorders, cognitive disorders, and anxiety [6,7].

The definition of fatigue, which is a subjective symptom, has been a source of controversy for many years. It has been variously defined as “tiredness”, “lack of energy”, “malaise”, “exhaustion”, and “motor weakness”. The Fatigue Guidelines Development Panel of the Multiple Sclerosis Council for Clinical Practice Guidelines defines fatigue as a “subjective lack of physical and/or mental energy that is perceived by the individual or caregiver to interfere with usual or desired activity”. Recently, some have called for the adoption of a different definition of fatigue put forth by Mills and Young, who defined it as “reversible motor and cognitive impairment, with reduced motivation and desire to rest” [3,8,9,10].

Fatigue is the most common symptom of patients with MS (PwMS), reported by approximately 75% at some point during the course of the disease. It represents one of the main causes of reduced quality of life among PwMS and is considered by some to be the most disabling symptom, in some cases surpassing motor deficit and pain. It interferes with the social activities of PwMS and is also associated with decreased work capacity, leading to job loss and therefore having a significant socioeconomic impact [3,11,12,13,14,15,16,17].

Despite recent studies on the topic, the pathophysiology of fatigue in PwMS is not yet fully understood. It is unanimously accepted that immunological factors are involved, but fatigue is also frequently associated with mood and sleep disorders, symptoms frequently reported in PwMS [3].

The main objectives of the study were to identify the sociodemographic determinants of fatigue in a cohort of Caucasian PwMS and also to evaluate the existing relationship between the presence and severity of fatigue and other neuropsychiatric manifestations of the disease (depression, cognitive disorders). Another objective was to examine how the sociodemographic determinants of fatigue differ between PwMS and healthy controls (HCs).

## 2. Materials and Methods

We conducted a cross-sectional study involving 150 consecutive patients diagnosed with RR or SPMS who were under the care of the Regional MS Center in Targu Mures. These patients had been previously diagnosed and were actively receiving disease-modifying therapies as part of the MS treatment protocol. We also included a control group of 100 HCs.

Inclusion criteria for the PwMS group were age > 18 years, signing the informed consent form, and an MS diagnosis according to the McDonald 2017 criteria [18]. The exclusion criteria were clinical recurrence or corticosteroid treatment within the last 30 days, diagnosis of other physical or psychiatric conditions that could influence the clinical evaluation or performance of neuropsychological tests, use of antidepressant or antipsychotic medications in the last two months, pregnancy, history of addiction to alcohol or other substances, mental retardation diagnosis, diagnosis of neoplasia or other autoimmune diseases, and participation in clinical trials with experimental therapies.

The inclusion criteria for the HC group were the absence of MS, signed informed consent form, and age > 18 years. The exclusion criteria for the HC group were almost similar to those applied to PwMS: the presence of any condition associated with a physical disability, diagnosis of a psychiatric condition, diagnosis of mental retardation, autoimmune diseases, or cancer.

All PwMS and HCs completed a questionnaire with demographic questions (age, sex, marital status, children, education level, occupational status, body mass index (BMI), and tobacco and alcohol consumption). For PwMS, questions related to their disease were also included (duration, treatment type, treatment duration, number of relapses, and MS form).

The degree of disability was evaluated using the Expanded Disability Status Scale (EDSS), a unified scale evaluating the level of neurological impairment in PwMS in eight functional systems [19].

Fatigue was assessed using one of the most suitable and appropriate tools for measuring the degree of fatigue: the Modified Fatigue Impact Scale (MFIS). The MFIS measures fatigue’s impact on normal and habitual physical, cognitive, and psychosocial functioning over the past four weeks. It comprises 21 questions, each answered with a value from 0 to 4. The scores for the physical (PS), cognitive (CS), and psychosocial (PSS) subscales and the total score are obtained by summing these values. The higher the score, the higher the level of fatigue and the greater its impact on the life of PwMS. A total score of ≤38 points is considered no fatigue impact, 39–58 points is considered a low fatigue impact, and ≥59 points is considered a high fatigue impact on the quality of life [17,20].

The level of depression was determined using the most frequently used depression assessment questionnaire in PwMS: the Beck Depression Inventory-II (BDI-II). The BDI-II comprises 21 questions assessing symptoms and behaviors suggestive of depression, such as feelings of guilt, pessimism, fatigue, and irritability; changes in sleep; and loss of appetite. Therefore, it assesses the somatic, cognitive, affective, and motivational symptoms characteristic of depression. Each question has four answers with scores from 0 to 3, giving a maximum score of 63. These scores can be used to define different degrees of depression, from mild to severe. A score of 11–16 indicates a mild mood disorder, 17–20 indicates borderline depression, 21–30 indicates mild depression, 31–40 indicates severe depression, and >40 indicates extreme depression [21].

Cognitive disorders were assessed using the Symbol Digit Modalities Test (SDMT), one of the most sensitive tests for measuring information processing speed and general cognitive impairment. This test comprises a set of symbols assigned numbers from 1 to 9 and nine rows of symbols. The patient must indicate the number corresponding to each symbol within 90 s. The SDMT’s primary outcome is the total sum of the digits correctly indicated next to each symbol; the higher the score, the better the information processing speed. The oral version of the SDMT was used, and the number of correct answers was recorded [22].

This study was performed according to the principles stated in the Declaration of Helsinki and approved by the local ethics committee.

After the PwMS and HCs signed the informed consent form, they completed the questionnaire with demographic questions. For the PwMS, data related to MS were obtained from their anamnesis and medical files. The MFIS, BDI-II, and SDMT were later administered to all study participants. A neurologist then evaluated the PwMS, and their EDSS score was calculated.

### Statistical Analysis

Differences between PwMS and HC regarding the outcome variables were described using standard statistics and evaluated for significance according to the empirical distribution toward normality probability. Normality was tested using the Kolmogorov-Smirnov test, quartile plots (Q-Q plots), skewness, and kurtosis. In the case of a normal distribution, we applied parametric tests such as the *t*-test. For non-parametric data, we used the Mann–Whitney U test and the Kruskal–Wallis test (with Dunn–Bonferroni post hoc correction).

Correlations between continuous variables were evaluated by calculating Pearson’s or Spearman’s coefficients, depending on whether the linearity assumption was satisfied. The coefficients were estimated with 95% confidence intervals and *p*-values. The Chi-Square test was used to determine whether there was a statistically significant association between two categorical variables. Linear univariate and multivariate regressions were employed to evaluate associations between fatigue and demographic and disease-related data in PwMS, considering the MFIS score as the dependent variable.

The statistical analysis was conducted using SPSS version 26, and GraphPad Prism version 9.3.0 was utilized for figure generation.

## 3. Results

### 3.1. Demographical and Clinical Data of the Patient and Control Groups

This study included 150 PwMS and 100 HCs. The demographic and disease-related data of the PwMS and HCs are presented in Table 1.

PwMS and HCs did not differ significantly in education level (*p* = 0.1100, chi-square test). Regarding marital status, the percentage of divorced individuals was higher in the PwMS group (8.7%) than in the HC group (1.0%). The percentage of individuals with children was significantly higher in the PwMS group (69.3%) than in the HC group (59%; *p* = 0.0100). However, BMI did not differ significantly between the PwMS and HC groups.

### 3.2. Modified Fatigue Impact Scale in Patient and Control Groups

**Figure 1:**
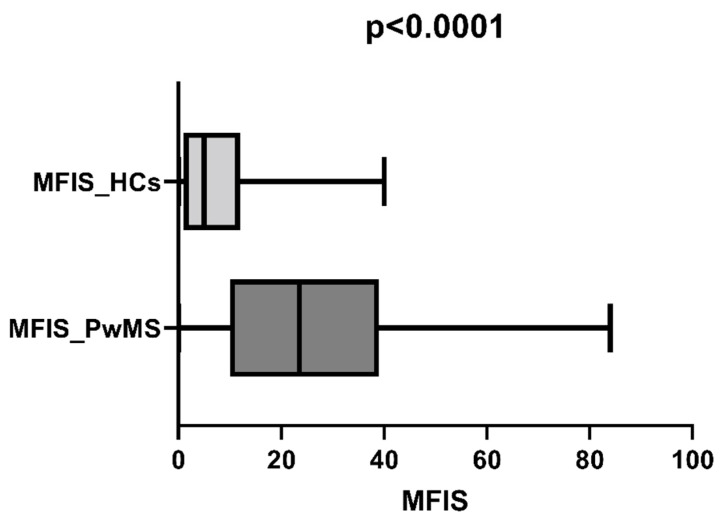
Comparison of fatigue levels between the PwMS and HC groups.

The median scores for the MFIS were calculated for each group and compared using the Mann–Whitney test to compare the fatigue levels of PwMS and HCs. The PwMS group had significantly higher MFIS scores than the HCs (*p* = 0.0001; Figure 1).

### 3.3. Relationship between Demographic and Disease-Related Factors and Fatigue

In order to determine the factors influencing the incidence and severity of fatigue in PwMS, their demographic and disease-related data were correlated, and their cognitive test and mood assessment results were correlated with their MFIS scores. Using Spearman’s rank correlation coefficient, we found that MFIS scores correlated positively with age, total number of relapses, total disease duration, EDSS score, and BDI-II score and negatively with cognitive performance. However, MFIS scores did not correlate with BMI in PwMS (Table 2).

In HCs, MFIS scores correlated positively with age and negatively with cognitive performance reflected by SDMT scores. Like in PwMS, MFIS scores did not correlate with BMI in HCs (Table 3).

The influence of patient sex on fatigue was statistically examined using the Mann–Whitney test. Fatigue levels were significantly lower among males than females PwMS (*p* = 0.0120). Regarding occupational status, fatigue levels were significantly lower among PwMS who worked full-time or were students than PwMS who worked part-time, were on a disability pension, or were retired (*p* = 0.0001). However, fatigue levels did not differ significantly by sex or occupational status among HCs.

Regarding marital status, PwMS who were unmarried but living with a partner had significantly lower fatigue levels than single, married, divorced, and widowed PwMS (*p* = 0.0070). In contrast, single HCs had the lowest fatigue levels, followed by unmarried HCs living with a partner and married HCs, with widowed or divorced HCs having the highest fatigue levels (*p* = 0.0040) (Table 4).

In both PwMS and HCs, those with children had significantly higher fatigue levels than those without children (*p* = 0.0180 and *p* = 0.0001, respectively). However, we consider it important to mention that PwMS with children were older than those without children (45.66 ± 7.95 vs. 34.7 ± 9.1), respectively, they had higher values on the BDI-II (11.72 ± 10.48 vs. 8.08 ± 7.47). However, fatigue levels were not significantly associated with smoking or alcohol consumption in PwMS or HCs.

### 3.4. Fatigue Level in MS Subgroups

**Figure 2:**
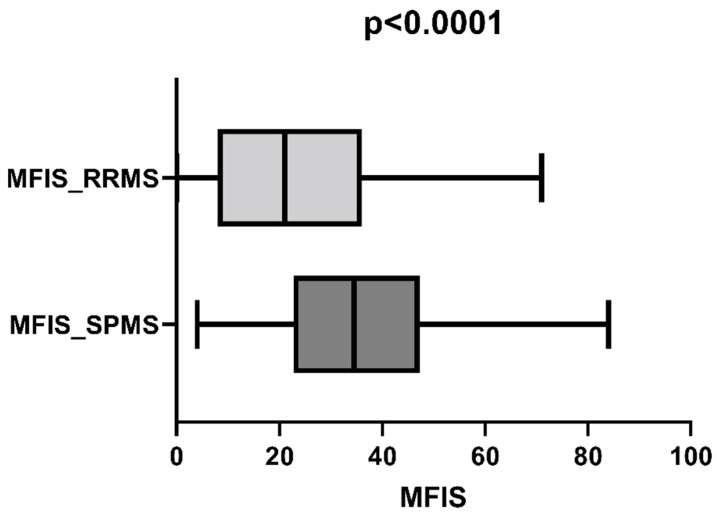
Comparison of fatigue levels between patients with RRMS and SPMS.

We compared fatigue levels between patients with RRMS and SPMS using the Mann–Whitney test to assess whether the clinical MS form influenced fatigue levels. Patients with RRMS had significantly lower fatigue levels than those with SPMS (*p* = 0.0010; Figure 2). Patients with SPMS had an older age compared to patients with RRMS (44.05 ± 8.33 vs. 41.81 ± 10.04) and also a higher level of physical disability (EDSS score of 3.49 ± 1.66 vs. 2.95 ± 1.96).

### 3.5. Relationship between Fatigue Level and Depression

BDI-II scores differed significantly between PwMS and HCs (*p* = 0.0001, chi-square test). While 90% of HCs and 58.7% of PwMS had normal results, 9% of HCs and 22% of PwMS had moderate mood disorders; 1% of HCs and 7.3% of PwMS had clinical depression; 0% of HCs and 6.7% of PwMS had moderate depression; 0% of HCs and 2.7% of PwMS had severe depression; and 0% of HCs and 2% of PwMS had extreme depression. Therefore, 18.7% of PwMS had clinically significant depression (BDI-II score ≥ 17; Table 5).

**Figure 3:**
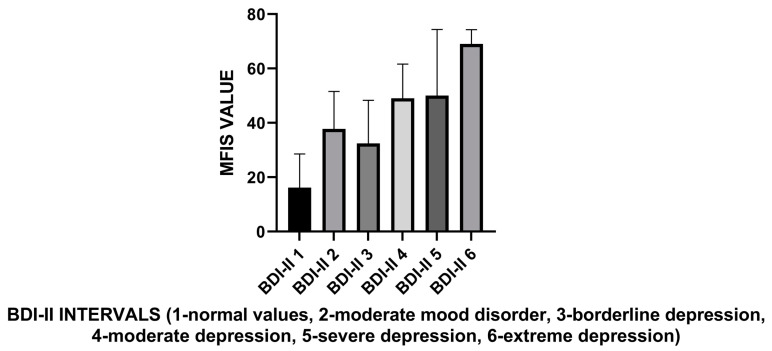
The relationship between the severity of fatigue and BDI-II test results.

Based on BDI-II score intervals, PwMS without mood disorders had significantly lower fatigue levels than those with depression (*p* = 0.0001; Figure 3).

Similarly, based on BDI-II scores, HCs with clinical depression had significantly higher fatigue levels than those with a normal BDI-II score (*p* = 0.0020).

### 3.6. Associations between Fatigue and Demographic and Disease-Related Data in the PwMS

Linear univariate and multivariate regressions were used to evaluate associations between fatigue and demographic and disease-related data in the PwMS, considering the MFIS score as the dependent variable. After multivariate analysis, their fatigue levels were influenced only by sex, occupational status, marital status, children status, and BDI-II test results (Table 6).

### 3.7. Analysis of Modified Fatigue Impact Scale Subscales

The scores for the three subscales of the MFIS test were also evaluated in PwMS and HCs. PwMS had significantly higher scores for all three MFIS subscales than HCs (*p* < 0.0001). In PwMS, PS, CS, and PSS scores were influenced by their degree of disability, cognitive performance, and mood disorders. While their age influenced the PS and PPS scores, it did not influence their CS scores (Table 7).

The scores for the three MFIS subscales were also evaluated by clinical MS form. Patients with RRMS had significantly lower PS, CS, and PSS scores than patients with SPMS (*p* < 0.0001).

In HCs, PS and CS scores were unaffected by age and SDMT and BDI-II scores, while PSS scores were only influenced by mood disorders (Table 8).

The scores obtained for the three subscales did not differ significantly by sex in HCs. However, female PwMS had significantly higher PS (*p* = 0.0100) and PSS (*p* = 0.0010) scores than male PwMS.

### 3.8. Modified Fatigue Impact Scale in Treatment Groups

**Figure 4:**
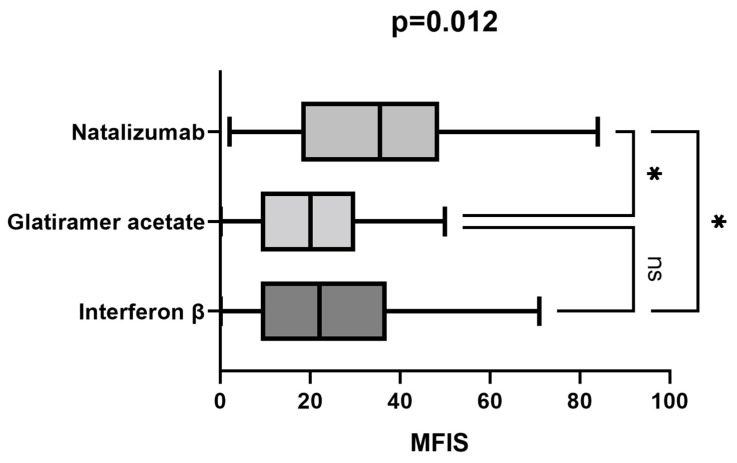
MFIS level in treatment subgroups. *—statistically significant.

We compared the MFIS scores among patients treated with interferon β, glatiramer acetate, and natalizumab. Patients treated with natalizumab presented significantly higher fatigue levels compared to the interferon β and glatiramer acetate treated groups (*p* = 0.03, *p* = 0.04, Figure 4). EDSS values were also significantly higher in the natalizumab-treated group compared to interferon β and glatiramer acetate groups (5.2 vs. 2.5, *p* < 0.001). There was no statistically significant age difference between treatment groups.

## 4. Discussion

Fatigue is one of the most common “silent” non-motor symptoms reported by PwMS, with a prevalence of 60–96%. Approximately 40% of PwMS reported fatigue as the most disabling symptom of their disease and the main cause of unemployment in PwMS. Some studies have shown that it can be the first MS symptom to manifest [8,9,17,23].

The etiology of fatigue in PwMS is multifactorial, with immunologic abnormalities recognized as a crucial factor and proposed as the primary mechanism. This hypothesis is supported by studies demonstrating that interferon-gamma and tumor necrosis factor-alpha are the most important mediators of fatigue in PwMS [3,24,25,26]. Some studies have also demonstrated an endocrine influence on fatigue. PwMS with fatigue have significantly lower levels of the hormone dehydroepiandrosterone than patients without fatigue. Another argument for this hypothesis is the improvement in fatigue under high-dose corticosteroid treatment [3,27,28]. Another primary mechanism involved in the pathophysiology of MS-associated fatigue is axonal loss, mainly in the frontal cortex and basal ganglia, as shown by non-conventional neuroimaging techniques [3,28]. Tartaglia et al. used functional magnetic resonance imaging to demonstrate an association between fatigue and increased recruitment of the bilateral cingulate gyri and left primary sensory cortex [29,30].

Secondary mechanisms considered responsible for fatigue development in PwMS are the severity of their physical disability, clinical MS phenotype, sleep disorders, and depression. Some of the therapies used to treat MS can also contribute to fatigue, such as some disease-modifying therapies (e.g., interferon-beta) and antispasmodic and anxiolytic therapies [3].

Fatigue is also influenced by disease evolution, and is more severe in periods of MS exacerbation. Physical exertion and heat exposure also play a role, with fatigue worsening during the day, especially in the summer months [16,23].

Fatigue is a complex symptom comprising three domains (physical, psychosocial, and cognitive). For this reason, self-rated questionnaires were developed to evaluate all three aspects of fatigue in PwMS. One of the most used of these is the MFIS, which comprises 21 questions [8,9]. The three domains of fatigue seem to have different determining factors. Ford et al. were the first to observe that mental but not physical fatigue strongly correlated with depression and anxiety [31]. Our study confirmed the published data: PwMS had significantly higher fatigue levels than HCs, reflected in their higher scores for the total MFIS and the PS, CS, and PSS subdomains [32].

Our study evaluated modifiable and non-modifiable determinants of fatigue in PwMS and HCs. Age was a non-modifiable determining factor of fatigue in both groups. However, sex influenced fatigue severity only in PwMS, with male PwMS having significantly lower fatigue levels than female PwMS. In HCs, fatigue levels did not differ significantly between males and females. When the sex and age of PwMS were examined as predictors of fatigue in the three MFIS subdomains, they influenced the PS and PSS but not the CS. Studies have reported contradictory findings on the influence of sex on fatigue. Trojan et al. reported no statistically significant difference in fatigue severity between female and male patients [32].

The exact reasons for the gender differences in the experience of fatigue in MS are not entirely understood; there are several factors that may contribute to higher fatigue levels in female MS patients compared to males. These factors include hormonal differences, sociocultural factors, immune system differences, and coexisting conditions. Fluctuations in hormone levels, particularly estrogen, can impact fatigue levels. Women experience hormonal changes throughout their menstrual cycle, during pregnancy, and in menopause, which can influence fatigue. Gender roles and societal expectations may also play a role. Women often take on more responsibilities related to family and caregiving, which can be physically and emotionally taxing, and this can contribute to fatigue. Additionally, women may be more likely to report fatigue and seek medical attention, leading to a perception of higher fatigue levels [33,34].

Weiland et al. [35] found that approximately two-thirds of 2.469 PwMS had clinically defined fatigue and evaluated the demographic and disease-related factors influencing its presence. Our group of PwMS, they found fatigue associated with advanced age and female sex, in addition to marital status, occupational status, children status, and total disease duration. Our study confirmed these results, with retired, disability pension, and working part-time PwMS having significantly higher fatigue levels than those who worked full-time. In the HC group, occupational status did not have an influence on the level of fatigue. These results can be explained by the fact that, in the case of PwMS, the primary reason for working part-time or being retired is the presence of physical disability, which renders them unable to work. Conversely, in the HC group, retired patients do not exhibit physical disabilities, as this is one of the inclusion criteria. When attempting to elucidate the variations in fatigue levels based on marital status, we observed that, aside from the age differences between these patient subgroups (where the married, divorced, and widowed patients were older than those living with a partner), the patients from the last subgroup had the lowest scores on the BDI-II. Although we anticipated that depression and fatigue might be more pronounced in divorced and widowed patients, we did not expect these symptoms to be more severe in married patients compared to those living with a partner or in patients with children compared to those without children. These differences are likely due to the diagnosis of a disease with an unpredictable course, a high risk of developing severe disability, and uncertainty about the future.

Regarding the disease-related data, as in Weiland et al. [35], fatigue correlated significantly with total disease duration and the degree of disability reflected by the EDSS score. The incidence and severity of fatigue were higher in patients with SPMS than with RRMS. This result of our study could also be explained by the fact that patients with SPMS had an older age and a higher degree of disability compared to patients with RRMS, variables that represent important independent determinants of fatigue. In addition, the total number of relapses during last year correlated positively with MFIS scores. Another study reported no statistically significant difference in fatigue severity between patients with RRMS and SPMS [32]. Our study observed different disease-related factors associated with the three MFIS subscales. Among patients with SPMS, those with a high degree of disability had significantly higher scores for all three MFIS subdomains. However, disease duration correlated only with the PS and PSS scores. Our results are consistent with other studies that found a strong association between fatigability and disability [36,37,38,39].

Regarding modifiable lifestyle factors, our study found alcohol consumption, smoking, and BMI not to be factors determining fatigue in PwMS or HCs, contradicting a previous Australian study [35]. These contradictory results could be explained by differences in lifestyle factors between the Australian study’s PwMS and HC groups and by the significantly smaller number of PwMS in the two studies. In our study, approximately half of the PwMS and HCs had never smoked, 80% of the PwMS did not consume alcohol, and half of the study participants had a normal BMI.

In our study, depression was a significant predictor of fatigue in both PwMS and HCs in univariate analyses and remained a contributor to fatigue in the multivariate regression models. In addition, depression was associated with increased scores for all three MFIS subdomains in PwMS and HCs. Other studies have reported similar results, suggesting that fatigue and depression share common mechanisms, such as psychosocial factors and brain lesions [39,40]. Considering that a strong association between fatigue and depression was also identified in our HC group, it is likely that the two share a common underlying mechanism linked especially to psychosocial factors or an interdependent relationship.

Cognitive impairment was a predictor of PS and CS fatigue in both PwMS and HCs but of PSS fatigue only in PwMS. Previous studies have found a significant relationship between fatigue and cognitive impairment spatially in the memory and attention domains, raising the hypothesis of a bidirectional relationship between the two symptoms: the presence of fatigue implies an increased level of attention and concentration for the performance of cognitive tasks, while attention disorders can interfere with the performance of some activities, resulting in increased fatigue [41,42,43,44,45].

Considering the increased prevalence of fatigue among PwMS and its significant negative impact on their quality of life, work capacity, and daily activities, identifying and treating fatigue is essential. The multifactorial etiology of fatigue makes its treatment challenging for clinicians. Currently, treatment for fatigue is limited to pharmacological therapy with antidepressants and wake-promoting agents, cognitive behavior therapy, exercise therapy, and cooling therapy [35,46,47,48]. However, mindfulness, exercise, and education programs have shown promising results [46,47,48,49].

Our study reinforced the view that, besides numerous sociodemographic and non-modifiable disease-related factors, fatigue is also associated with diverse modifiable factors. Regardless of fatigue’s pathophysiological mechanism, identifying these potentially modifiable factors should be valuable for clinicians because their control could be associated with a significant decrease in the fatigue level of PwMS. The severity of fatigue could be significantly affected by the early identification of conversion from RRMS to SPMS and escalation of disease-modifying therapy, identification of depression and institution of appropriate antidepressant treatment, and identification of cognitive disorders and initiation of cognitive behavioral therapy.

In relation to differences in fatigue levels depending on the choice of disease-modifying therapy, we observed that patients treated with natalizumab had significantly higher MFIS scores compared to those receiving interferon β and glatiramer acetate. Natalizumab is known for its effectiveness in managing disease activity in PwMS. However, it is important to acknowledge that there are patients who exhibit a suboptimal response to natalizumab and describe a phenomenon known as “wearing off”. A study conducted by Bringeland GH et al. revealed that patients experiencing the “wearing off” phenomenon reported higher levels of fatigue compared to those without this phenomenon. One limitation of our study is that we did not assess the presence of the “wearing off” phenomenon in patients treated with natalizumab. Additionally, another possible explanation for the higher MFIS scores among patients treated with natalizumab could be attributed to the significantly higher degree of disability observed among patients in this treatment group [50].

The main limitations of our study are its relatively small number of participants and the possibility that the data it collected may have been influenced by responder bias, especially the answers related to lifestyle (alcohol consumption and smoking). Another limitation of the study is the fact that patients with primary progressive multiple sclerosis were not included because, at the time of the study in our country, ocrelizumab was not reimbursed for the treatment of primary progressive multiple sclerosis. Nevertheless, our study is strengthened by its evaluation of associations between fatigue and various disease-related factors (duration, number of recurrences, and degree of disability), mood disorders, and cognitive performance in addition to demographic factors.

## 5. Conclusions

Our study revealed a strong correlation between the severities of fatigue, overall and for each MFIS subdomain, and depression (cognitive disorders) in PwMS and HCs. The other factors determining fatigue in PwMS were their degree of disability and disease duration. Considering the significant impact of fatigue on the quality of life of PwMS, clinicians must diagnose fatigue in PwMS as early as possible, identify its modifiable determinants, and manage it effectively to increase their quality of life.

## Figures and Tables

**Table 1:** Descriptive statistics of the demographic data of PwMS and HCs and disease-related data of PwMS.

	PwMS N = 150	HCs N = 100	Statistical Significance
Current age (Mean ± SD)	42.32 ± 9.66	41.53 ± 12.17	NS
Gender (Male/Female)	53:97	41:59	NS
Age at disease onset (Mean ± SD)	31.56 ± 8.75		NA
Duration of the disease—years (Mean ± SD)	10.85 ± 6.44		NA
Annual relapse rate—last year (Mean ± SD)	0.58 ± 1.06		NA
MS Form (RRMS:SPMS)	116:34		NA
EDSS (Mean ± SD)	2.87 ± 1.97		NA
BDI-II (Mean ± SD)	10.6 ± 9.7	4.56 ± 4.31	*p* < 0.0001
MFIS total score (Mean ± SD)	26.22 ± 18.69	8.02 ± 9.16	*p* = 0.0001
MFIS-Physical subscale (Mean ± SD)	15.21 ± 9.74	4.68 ± 5.43	*p* < 0.0001
MFIS-Cognitive subscale (Mean ± SD)	8.29 ± 9.01	3 ± 3.84	*p* < 0.0001
MFIS-Psychosocial subscale (Mean ± SD)	2.75 ± 2.39	0.7 ± 1.22	*p* < 0.0001
SDMT (Mean ± SD)	30.84 ± 12.82	40.41 ± 12.58	*p* < 0.0001
BDI (Mean ± SD)	22.1 ± 21.29	24.29 ± 20.7	*p* < 0.0001

NS—not significant, NA—not applicable.

**Table 2:** Correlations between MFIS scores and age, BMI, disease-related data (duration, number of relapses, and EDSS), and SDMT and BDI-II scores in PwMS.

		SDMT	Current Age	BMI	Total Number of Relapses	Disease Duration	EDSS	BDI-II Scores
Total MFIS Score	r	−0.312	0.280	0.043	0.314	0.221	0.444	0.718
	*p* value	<0.0001	**0.001**	0.600	**<0.0001**	**0.007**	**<0.0001**	**<0.0001**
	N	150	150	150	150	150	150	150

**Table 3:** Correlations between MFIS scores and age, BMI, and SDMT and BDI-II scores in HCs.

		SDMT	Current Age	BMI	BDI
Total MFIS Score	r value	−0.282	0.476	0.181	0.71
	*p* value	**0.004**	**<0.0001**	0.071	**<0.0001**
	N	100	100	100	100

**Table 4:** Age and BDI-II score according to marital status.

	Single	Married	Living with a Partner	Divorced	Widowed
**Current age (Mean ± SD)**	36 ± 11.47	44.56 ± 8.19	30.5 ± 7.12	43.3 ± 5.12	55.33 ± 8.02
**BDI-II Score (Mean ± SD)**	9.8 ± 8.44	10.85 ± 10.15	3.5 ± 3.06	13.15 ± 11.08	17.66 ± 5.03

**Table 5:** Comparison of BDS-II intervals between PwMS and HCs.

*p* < 0.0001			Groups		Total
			HCs	PwMS	
BDI-II Interval	1	Count	90	88	178
		%	90.0%	58.7%	71.2%
	2	Count	9	33	42
		%	9.0%	22.0%	16.8%
	3	Count	1	11	12
		%	1.0%	7.3%	4.8%
	4	Count	0	10	10
		%	0.0%	6.7%	4.0%
	5	Count	0	4	4
		%	0.0%	2.7%	1.6%
	6	Count	0	3	3
		%	0.0%	2.0%	1.2%
Total		Count	100	150	250
		%	100.0%	100.0%	100.0%

Legend: 1, BDI-II scores of 0–10 (normal); 2, BDI-II score of 11–16 (moderate mood disorders); 3, BDI-II score of 17–20 (borderline depression); 4, BDI-II score of 21–30 (moderate depression); 5, BDI-II score of 31–40 (severe depression); 6, BDI-II score of >40 (extreme depression).

**Table 6:** Linear regression analysis of associations between fatigue and demographic and disease-related data in PwMS.

**Univariate Linear Regression Model**							
**Model**	**Unstandardized Coefficients**		**Standardized Coefficients**	**t**	***p* Value**	**95.0% Confidence Interval for B**	
	**B**	**Std. Error**	**Beta**			**Lower Bound**	**Upper Bound**
Onset symptom	0.248	0.970	0.021	0.255	0.799	−1.670	2.165
Type of MS	6.935	1.737	0.312	3.992	0.0001	3.502	10.369
Age at disease onset	0.203	0.174	0.095	1.165	0.246	−0.141	0.547
Total number of relapses	0.986	0.243	0.316	4.052	0.0001	0.505	1.466
Total disease duration	0.740	0.231	0.255	3.211	0.002	0.285	1.196
EDSS	4.097	0.700	0.434	5.854	0.0001	2.714	5.480
BDI-II scores	1.542	0.090	0.736	17.104	0.0001	1.365	1.720
Sex	5.649	2.324	0.153	2.431	0.016	1.072	10.226
Occupational status	1.293	0.696	0.116	1.856	0.065	−0.079	2.665
Marital status	2.226	1.131	0.134	1.969	0.050	0.000	4.453
Presence of children	6.364	2.250	0.177	2.828	0.005	1.931	10.796
Dependent Variable: Total MFIS Score							
**Multivariate Linear Regression Model**							
**Model**	**Unstandardized Coefficients**		**Standardized Coefficients**	**t**	***p* Value**	**95.0% Confidence Interval for B**	
	**B**	**Std. Error**	**Beta**			**Lower Bound**	**Upper Bound**
Type of MS	1.253	1.961	0.056	0.639	0.524	−2.625	5.130
Total number of relapses	0.110	0.215	0.035	0.511	0.610	−0.315	0.534
Total disease duration	0.133	0.202	0.046	0.660	0.510	−0.266	0.532
EDSS	1.356	0.922	0.144	1.472	0.143	−0.466	3.178
BDI-II scores	1.180	0.123	0.617	9.567	0.0001	0.936	1.424
Sex	5.293	2.269	0.143	2.332	0.020	0.823	9.762
Occupational status	1.293	0.696	0.116	1.856	0.045	−0.079	2.665
Marital status	2.226	1.131	0.134	1.969	0.045	0.000	4.453
Presence of children	9.023	2.445	0.251	3.690	0.0001	4.207	13.839
Dependent Variable: Total MFIS Score							

**Table 7:** Correlations between PwMS characteristics and MFIS subscale scores.

	Physical Subscale	Cognitive Subscale	Psychosocial Subscale
Age	r = 0.288	r = 0.1558	r = 0.321
	*p* = 0.0003	*p* = 0.057 (NS)	*p* < 0.0001
EDSS	r = 0.595	r = 0.262	r = 0.516
	*p* < 0.0001	*p* = 0.0012	*p* < 0.0001
BDI	r = 0.608	r = 0.648	r = 0.613
	*p* < 0.0001	*p* < 0.0001	*p* < 0.0001
SDMT	r = −0.316	r = −0.282	r = −0.241
	*p* < 0.0001	*p* = 0.0005	*p* = 0.00029

**Table 8:** Correlations between HC characteristics and MFIS subscale scores.

	Physical Subscale	Cognitive Subscale	Psychosocial Subscale
Age	r = 0.448	r = 0.416	r = 0.09
	*p* < 0.0001	*p* < 0.0001	*p* = 0.33 (NS)
BDI	r = 0.709	r = 0.575	r = 0.326
	*p* < 0.0001	*p* < 0.0001	*p* = 0.0009
SDMT	r = −0.269	r = −0.28	r = −0.04
	*p* = 0.0067	*p*= 0.0045	*p* = 0.63 (NS)

## Data Availability

Data are available from the corresponding author upon reasonable request.
